# Assessment of focused ultrasound stimulation to induce peripheral nerve activity and potential damage *in vivo*

**DOI:** 10.3389/fneur.2024.1346412

**Published:** 2024-02-28

**Authors:** Bruno Rodríguez-Meana, Eva Santos-Nogueira, Sònia Trujillo-Vázquez, Anette Jakob, Esther Udina, Marc Fournelle, Xavier Navarro

**Affiliations:** ^1^Department of Cell Biology, Physiology and Immunology, Institute of Neurosciences, Universitat Autònoma de Barcelona, Bellaterra, Spain; ^2^Centro de Investigación Biomédica en Red de Enfermedades Neurodegenerativas (CIBERNED), Bellaterra, Spain; ^3^Fraunhofer IBMT, Sulzbach, Germany; ^4^Institute Guttmann of Neurorehabilitation, Badalona, Spain

**Keywords:** electrophysiology, ultrasound, nerve interface, nerve damage, nerve stimulation, sciatic nerve

## Abstract

**Introduction:**

Peripheral neuroprostheses are aimed to restore loss of sensory and motor functions by interfacing axons in the peripheral nerves. Most common interfaces in neuroprostheses are electrodes that establish electrical connection with peripheral axons. However, some challenges arise related to long-term functionality, durability, and body response. Recently, focused ultrasound stimulation (FUS) has emerged as a non-invasive approach to modulate the nervous system. However, it is controversial whether FUS can induce axon depolarization.

**Methods:**

We have assessed FUS applied in vivo to the rat peripheral nerve, with two objectives: first, to test whether FUS activates peripheral nerves under different stimulation conditions, and second, to evaluate if FUS inflicts damage to the nerve. FUS was delivered with three ultrasound transducers (Sonic Concept H115, H107, and H102) covering the largest set of parameters examined for FUS of peripheral nerves so far.

**Results:**

We did not obtain reliable evoked action potentials in either nerves or muscles, under any FUS condition applied, neither over the skin nor directly to the nerve exposed. Additional experiments ex vivo and in vivo on mice, confirmed this conclusion. When FUS stimulation was applied directly to the exposed sciatic nerve, neuromuscular function decreased significantly, and recovered one week later, except for FUS at 0.25 MHz. Histologically, degenerating nerve fibers were observed, with a tendency to be higher with the lower FUS frequency.

**Discussion:**

Past reports on the ability of ultrasound to stimulate the peripheral nerve are controversial. After testing a wide range of FUS conditions, we conclude that it is not a reliable and safe method for stimulating the peripheral nerve. Special consideration should be taken, especially when low-frequency FUS is applied, as it may lead to nerve damage.

## Highlights

- Pulsed FUS stimulation at 0.25, 0.5, 1.12, 1.63, and 3.58 MHz, using parameters ranging from 1 to 10 pulses of 1 to 200 ms and 0.1 to 5 MPa, did not produce excitation of peripheral nerve axons in vivo in the sciatic nerve of the rat.
- FUS stimulation applied on the plantar skin did not evoke electrophysiological activity in the sciatic nerve fibers of the rat. US of low frequency at high intensity evoked pain responses.
- When FUS stimulation was applied directly to the exposed sciatic nerve, the amplitude of electrical evoked responses decreased significantly after using the transducers with the frequencies of 0.25, 0.5 and 1.12 MHz.
- Light microscopy of transverse sections of the nerve distal to the application of the FUS, showed degenerating fibers, mainly localized at the periphery of the nerve. The amount of axonal degeneration was larger with the lower US frequency.

## 1 Introduction

Damage to the peripheral nervous system (PNS) or limb amputations produce severe functional deficits in the affected subjects. In such cases, peripheral neuroprostheses can re-establish sensory and motor activity by directly stimulating peripheral axons, or by replacing injured nerves, connecting proximal nerve segments with denervated muscles or artificial prostheses (1). Electrodes that contact peripheral nerves using an electrical coupling method are the type of interfaces most commonly used in neuroprostheses. Over the last decades, neural interfaces have evolved to optimally record and/or selectively stimulate peripheral axons, and they can be implanted around or within the peripheral nerves (2, 3). Extraneural interfaces, such as nerve cuff electrodes, are placed around the nerve, whereas intraneural electrodes are implanted longitudinally or transversally within the nerve fascicles. Despite their invasiveness, the latter need lower threshold to stimulate the axons, higher selectivity, and have lower signal-to-noise ratio for recording than extraneural electrodes (4, 5).

However, a major problem that implantable interfaces face to be functional over time relies on their robustness, biocompatibility and body rejection. Any implanted device is accompanied by some degree of tissue injury, which induces an immune reaction (6) leading to possible nerve damage and subsequent development of fibrotic tissue and encapsulation. As a result, the electrode performance declines over time, requiring an increased stimulation threshold, together with decreasing the stability of the recording signals (7–10). Thus, efforts are taken to diminish the foreign body reaction and connective encapsulation, by developing minimally or non-invasive interface systems which do not directly interact with the nervous tissue, thus avoiding the immune response (2, 11).

There are other possible methods of coupling neuroprostheses to the PNS depending upon the type of biophysical signal conveyed. Within these technologies, focused ultrasound stimulation (FUS) has won attention as a therapeutic intervention to activate or modulate the nervous system. FUS uses a transducer with an acoustic lens to focus a sound wave to a defined focus spot within the body. This technique utilizes the ability of US to generate heat or mechanical vibration to degrade target elements inside the body, such as tumors or fibrotic tissue, without damaging adjacent tissues.

Under appropriate stimulation intensities, the high precision and non-invasiveness of FUS has been applied in attempts to activate or block peripheral nerve activity (12–18). Although the mechanism for evoking axon depolarization remains unknown, it is hypothesized that FUS may directly open ion channels mechanically (19), induce energy transduction mechanisms, like intra-membrane bubble cavitation (20), or induce force membrane tension (21). It has also been reported that FUS activates specific ion channels, such as PIEZO2 (mechanosensitive), TRP family (mechanical or thermal sensitive), and TREK potassium channels (15, 22, 23), generating action potentials in both myelinated (Aβ and Aδ) and unmyelinated (C) fibers. Nevertheless, direct excitation of peripheral nerves with US remains controversial with opposite results reported. On one hand, *in vivo* and *ex vivo* experiments have shown that US can both excite and inhibit rodent nerves noninvasively (12, 24–26), and when applied directly to the sciatic nerve (17, 27). On the other hand, other studies have reported the inability of US to elicit compound nerve action potentials (CNAPs) in nerves both *in vivo* (14) and *ex vivo* (28), or even reduction of nerve excitability (14).

These discrepancies have engaged us to study whether the peripheral nerve can be activated with FUS stimulation, and thus, if it can be used as an interfacing system for neuroprostheses. We have employed our experimental *in vivo* electrophysiological set up in which we can effectively assess the stimulation capabilities of neural interfaces in the peripheral nerve (4, 5, 29, 30), and performed a follow-up functional and histological evaluation for assessing potential tissue damage and safety.

## 2 Materials and methods

All procedures were performed following protocols approved by the Ethical Committee of the Universitat Autònoma de Barcelona, in accordance with the European Communities Council Directive 2010/63/EU. Twenty-two Sprague–Dawley 12-week-old rats (10 male and 12 female) and 4 Balb/c 10-week-old (2 male and 2 female) mice were used. Animals were kept on standard laboratory food and tap water *ad libitum* with a light–dark cycle of 12 h. Animals were anesthetized with ketamine and xylazine (90/10 mg/kg, i.p.) for all stimulation experiments explained below.

### 2.1 Study design

**Figure 1:**
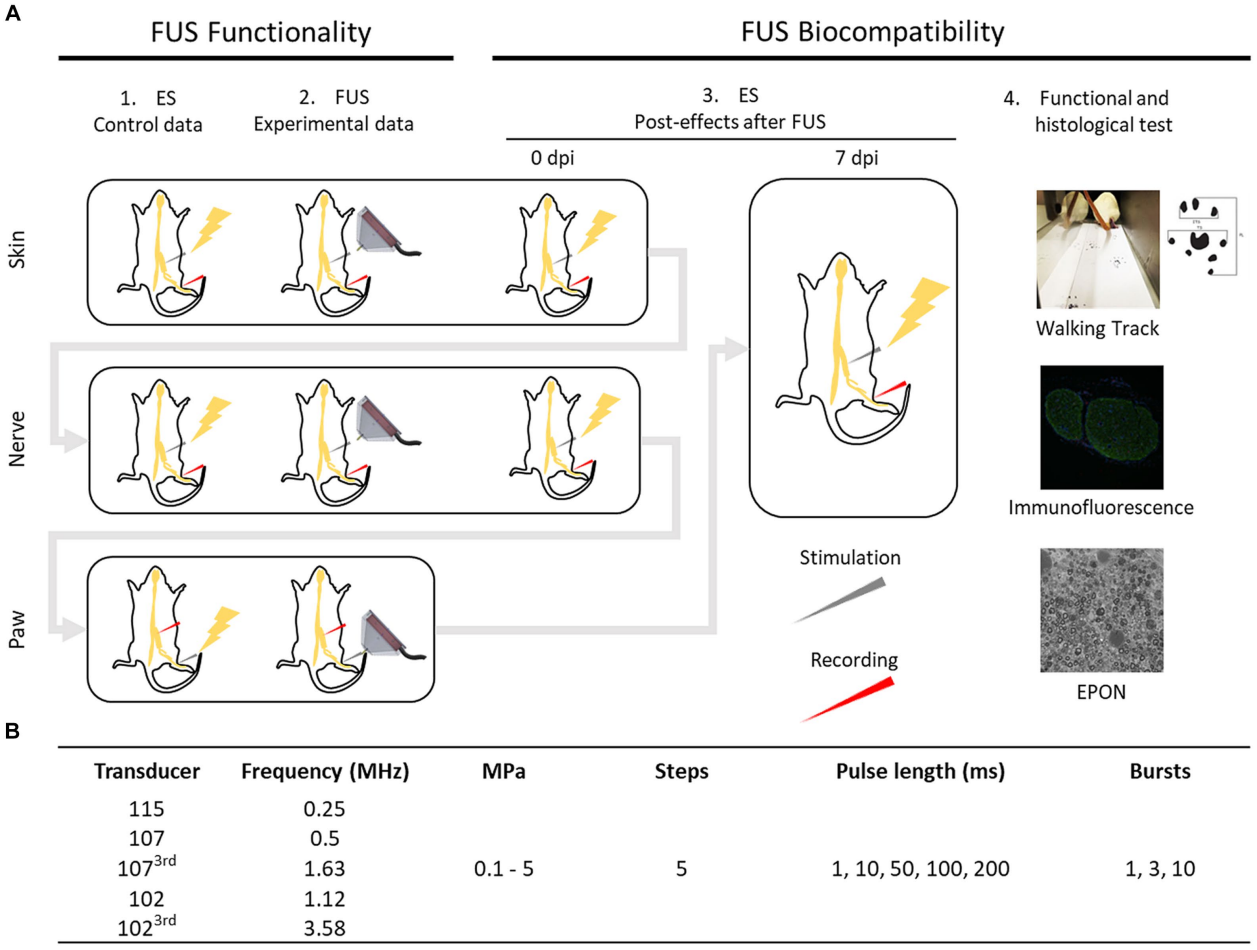
Schematic diagram of the design of the study **(A)**. ES: electrical stimulation. dpi: days post injury. FUS protocol applied with each transducer **(B)**.

The design of the full study is summarized in Figure 1. In each experimental session a defined protocol of stimulation with a given FUS transducer was applied to one rat. For each frequency tested 4 rats (2 males and 2 females) were used, and groups defined according to the FUS transducer frequency applied (Table 1). Control baseline electrophysiological values were first obtained with standard nerve conduction tests using electrical stimulation. Then, FUS was first applied over the skin overlying the sciatic nerve using a FUS frequency; at the end electrical stimulation was applied again to control nerve function properties. After ten minutes resting, the skin and muscle were sectioned to expose the sciatic nerve, and the same FUS stimulation protocol applied, followed by electrical stimulation. Finally, the FUS transducer was focused on the sole of the hindpaw. Altogether, in this study we investigated the largest set of frequencies and parameters and stimulation conditions that has been examined for US stimulation of peripheral nerves so far. We have also attempted to reproduce the settings of other reports in the literature using US for activating the peripheral nerve. We applied pressures below and above levels recommended in the FDA guidance for diagnostic ultrasound. After 7–9 days of the session, functional and conduction tests were conducted on each rat, and the sciatic nerve was harvested and processed for histological analyses to assess for potential damage to the nerve.

**Table 1:** US transducers characteristics.

Transducer model	H115	H107	H102	H107 – 3rd harmonic	H102 – 3rd harmonic
Center frequency (MHz)	0.25	0.5	1.12	1.63	3.58
Focal width (mm)	6.0	3.0	1.4	0.9	0.4
Focal length (mm)	39.5	21.4	10.0	7.0	3.2

Additional experiments were conducted with an *ex vivo* recording setup. The sciatic–tibial nerve of one female rat was harvested and placed longitudinally on a 15-electrode grid chamber for recording CNAPs. The nerve was kept moistened in Ringer-lactate solution. FUS stimulation was delivered from the three transducers focused on the nerve at one end, whereas electrodes placed >3 cm were used for recording.

Finally, another experiment with a female rat was carried out to elucidate the origin of some signals recorded in the muscles during nerve FUS stimulation. Briefly, the sciatic nerve was directly stimulated with FUS. After verifying the recording of signals, the nerve was dissected and removed from the animal. Then, FUS stimulation was repeated, maintaining the same transducer position but in the absence of the nerve, to investigate whether recorded signals were nerve originated or artefactual.

### 2.2 FUS devices

FUS was delivered with three commercial ultrasound transducers (Sonic Concept Model H115, H107, and H102) (Supplementary Figure S1). Models H107 and H102 work both at fundamental and at 3rd harmonic resonance, so five different center frequencies were tested (Table 1). The frequencies were chosen to cover the low frequency range from 0.25 to 3.5 MHz, generally used in studies for US Neuromodulation (31, 32). Each transducer worked with a radio frequency impedance matching network to match the transducer impedance to the impedance of the power source. Driving signals were delivered and amplified by an amplifier/generator radio frequency power source (AG Series Amplifier, model AG 1016) of 600 W. A software (SOMA software) was developed by Fraunhofer IBMT to control the generation of the FUS signals, allowing to adjust the different FUS settings and parameters. A manual axis positioner with a micromanipulator allowed easy positioning of the transducer over the animal. A laser beam was used to visualize the focus axis of the transducer to help position the focus at the site of interest. The cone of the transducer was filled with degassed water and the tip was sealed with a thin transparent membrane (Sonic Concepts, 2,214,017). To ensure the continuous coupling of the transducers tip to the target surface, a good amount of coupling gel was always used.

The transducer calibration was performed in a tank filled with deionized water under free field conditions using a hydrophone (HNR 0500, Onda Corp.). Pressure fields were recorded at different spatial locations, for verifying the intended focus of the transducer. The maximum pressure was detected when the hydrophone was located just in front and between 0 and 0.5 mm from the tip of the cone for all transducers and frequencies used.

### 2.3 Protocol of FUS stimulation

Three FUS stimulation settings were tested. First, the sciatic nerve was non-invasively stimulated through the skin. For this purpose, the skin over the sciatic nerve was shaved with depilatory cream. The laser beam was focused on the skin over the trajectory of the nerve guided by anatomical landmarks. The second was an invasive approach in which FUS stimulation was applied directly on the exposed sciatic nerve. In this case, the nerve was surgically exposed at the mid-thigh and carefully freed from adherences to surrounding tissues, and the wound maintained open with mini-retractors. A rectangular piece of Parafilm was carefully placed under the exposed nerve to isolate it from the surrounding muscles and tissues. The FUS focus was aligned on the nerve at the midthigh level by using the laser pointer. Electrophysiological signals were simultaneously recorded from both muscles and nerve, following previously reported methodology (4, 5, 33). Compound muscle action potentials (CMAPs) from the interosseus plantar (PL) and tibialis anterior (TA) muscles were recorded by means of monopolar needle electrodes inserted at the belly of each muscle. For recording compound nerve action potentials (CNAPs), the needle electrodes were placed near the lateral plantar nerve (LPN).

The third and last approach consisted in the FUS stimulation of the hindpaw, at the mid of the plantar side. The laser beam was adjusted to the point between the mid plantar pads. In this case, hook electrodes attached to the sciatic nerve were used for recording CNAPs and needle electrodes placed on the TA muscle for recording EMG signals. Also, in an effort to detect very low signals, tests were also performed using a polyimide cuff electrode placed around the sciatic nerve (34), and with the nerve desheathed and the spread fibers placed on a bipolar electrode to increase the number of axons in contact with the recording electrode.

Given that other authors who reported peripheral nerve activation induced by stimulation with FUS have used mice (12, 15, 24), the same three FUS stimulation approaches were used also on mice.

A fixed protocol of FUS parameters covering all settings tested in the literature was employed for the three FUS stimulation approaches: 5 sequences of 1, 3 or 10 pulses of 1, 10, 50, 100 or 200 ms duration, with an interstimulus interval of 100 ms. Intersequence interval was 1 s and pulse intensities increased from 0 to 5 MPa (effective pressure) on each sequence (0, 1.25, 2.5, 3.75 and 5 MPa) (Supplementary Table S1). The stimulus order was typically from less to more pulses, from short to long duration and from low to high intensity. The full protocol for one FUS frequency was applied to each rat during one session.

Electrical stimulation was used to control for good positioning of the recording electrodes, for comparison of the FUS and electrical induced responses, and to control for possible tissue damage or functional loss after nerve exposure to FUS. The sciatic nerve was electrically stimulated through a pair of monopolar needles percutaneously inserted at the sciatic notch. The paw was also stimulated using needle electrodes touching the plantar skin. Stimulation was provided by a Grass S44 stimulator and single square electrical pulses of 0.05 ms duration and up to supramaximal intensity were applied, for obtaining recruitment curves of the CMAPs and CNAP (4).

The electrophysiological signals were amplified by x200 or x500 for CMAPs and x1000 for nerve signals (P511AC amplifiers, Grass, West Warwick, RI, United States), and band-pass filtered (5 Hz to 2 kHz). Digital sampling of the signals was made with a PowerLab recording system (PowerLab16SP, ADInstruments, Bella Vista, Australia) at 20 kHz, and fed into LabChart7 software. The latency to the onset and the maximal baseline to peak amplitude of the evoked signals were measured.

During the session, animal body temperature was maintained constant using a thermostatic heating pad, anesthesia maintained by repeating anesthetic mixture injection, and mineral oil regularly poured on the wound to avoid tissue dryness.

### 2.4 Evaluation of nerve damage

After each FUS stimulation set, electrophysiological tests with electrical stimulation were performed to test that the nerve was functional. In addition, 9 days after the complete FUS stimulation session, nerve conduction tests and functional tests were conducted to assess functionality of the sciatic nerve. The Walking Track test was carried out to assess the locomotor function. The plantar surface of the rat hindpaws was painted with ink and the rat left to walk along a corridor with white paper on the base. The print length (PL), the distance between first and fifth toes (TS) and between second to fourth toes (IT) were measured on footprints of the operated and intact paws, and used to calculate the Sciatic Functional Index (SFI) (33).

For histomorphological analysis, the FUS exposed sciatic nerves were removed after the functional tests. Rats were euthanized with sodium pentobarbital (200 mg/kg i.p.), and the nerve harvested. A segment of the nerves, taken about 1 cm distal to the site where the US focus had been applied at the midthigh level, was fixed in 3% paraformaldehyde and 3% glutaraldehyde in 0.1 M sodium cacodylate buffer, pH 7.3 for 24 h at 4°C. Then, nerves were washed three times, postfixed for 1 h with 1% OsO_4_ in 0.1 M sodium cacodylate buffer, washed again in cacodylate buffer, dehydrated with ethanol/acetone and embedded in Epon. Semithin sections were cut with a diamond knife at 0.5 μm thickness, mounted on glass slides and stained with toluidine blue. Using an Olympus microscope image montages of the cross-section of each nerve were taken with a digital camera. Another segment of the distal sciatic nerves was fixed in 4% paraformaldehyde, cryoprotected in 20% sucrose in PBS, and processed for immunohistochemical labeling of axons (antibody RT97, against Neurofilament 200), and of macrophages (ionized calcium-binding adapter molecule 1 (Iba1)). Samples were washed and incubated with secondary antibodies Alexa Fluor 488 goat anti-chicken (1:200; A11039-Invitrogen), and Alexa Fluor 594 goat anti-rabbit (1:200; A21207-Invitrogen). Finally, sections were cover-slipped with Fluoromount containing DAPI (1:10000; Sigma-Aldrich). Sections were visualized with an epifluorescence microscope (Olympus BX51).

### 2.5 Data analysis

Grouped data are expressed as mean ± SEM. For comparison of changes over time, the results were normalized as percentage of the baseline value. Statistical comparisons were made with two-way ANOVA, and Bonferroni post-hoc test.

## 3 Results

### 3.1 *In vivo* FUS stimulation of peripheral nerves did not elicit any electrophysiological response

**Figure 2:**
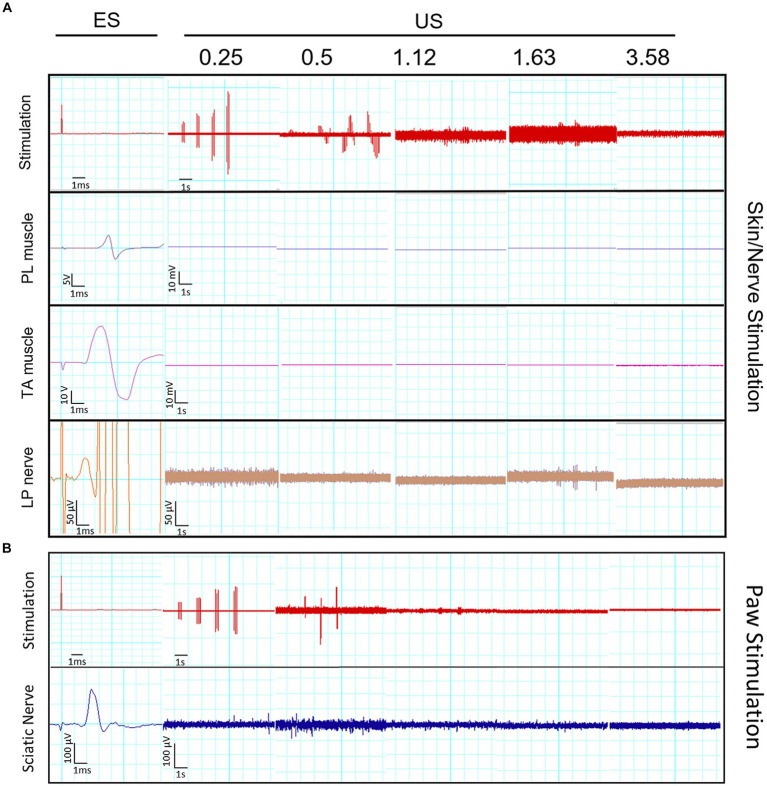
**(A)** Representative electrophysiological recordings of CMAPs recorded on plantar interosseus (PL) and tibialis anterior (TA) muscles and of CNAP recorded near the lateral plantar nerve (LPN), evoked by electrical stimulation (ES) of the rat sciatic nerve, and in response to US stimulation delivered with the different transducers at the indicated US frequency. For each FUS frequency five bursts were applied at 0, 1.25, 2.5, 3.75 and 5 MPa. The top trace is of the stimulation pulses; note that at US frequency higher than 0.25 the pulses are not recorded adequately since they overpass the sampling rate. Note the change in amplitude and time scales between recordings with ES and with US. **(B)** The bottom panel shows representative recordings of CNAP in the sciatic nerve evoked by ES of the LPN at the side of the hindpaw, and US stimulation with the different transducers focused on the lateral side of the hindpaw.

**Figure 3:**
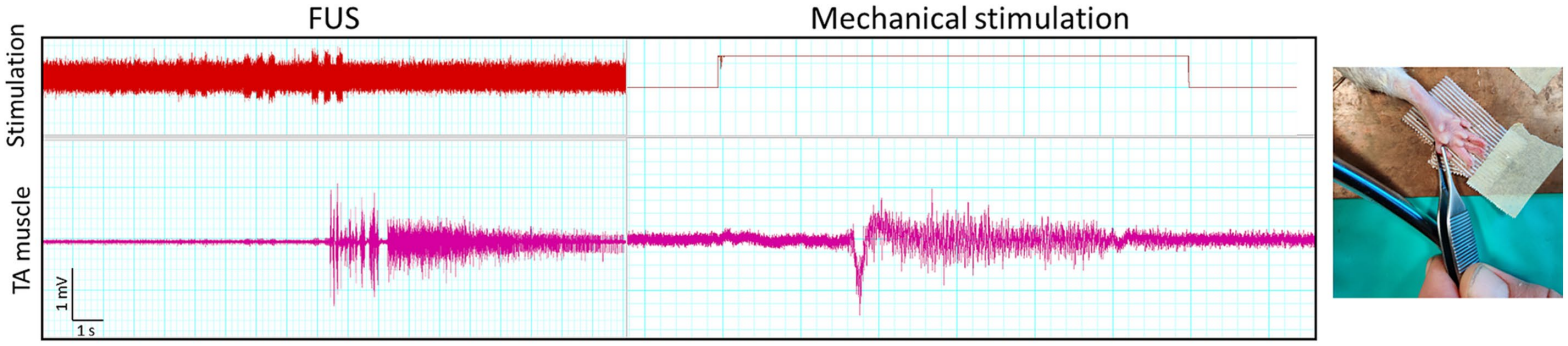
Recordings of withdrawal responses, evidenced by motor unit action potentials activated in the TA muscle, in response to mechanical pinching the toe and to US stimulation on the paw plantar surface at a 0.25 MHz US frequency.

Prior to FUS exposure, electrical stimulation of the sciatic nerve at the sciatic notch generated CMAPs of maximal amplitude of 8.0 ± 2.5 mV at the PL muscle and 50.8 ± 11.2 mV at the TA muscle, and CNAPs of 56.9 ± 31.1 μV from the LPN (Figure 2). However, when muscle and nerve activity was recorded in response to pulsed FUS stimulation at 0.25, 0.5, 1.12, 1.63 and 3.58 MHz, using parameters ranging from 1 to 10 pulses of 1 to 200 ms and 0.1 to 5 MPa, no consistent electrophysiological responses were recorded in any of the used setups (Figure 2). Our results indicate that low-intensity US stimulation is not able to directly excite the peripheral nerves, including myelinated motor (Aα) and sensory axons (Aαβ), at least for the wide range of parameters tested in this study. In addition, indirect evidence points that FUS were not able to activate smaller nociceptive axons (Aδ and C), since no consistent reflex responses were recorded (33, 35). Moreover, we could not elicit any CNAP, even if recording with a cuff or with the spread nerve, when applying the FUS focused on the plantar skin *in vivo*, indicating that terminal mechanoreceptors were not excited. For the transducer H115, mild withdrawal responses were elicited in some cases at the highest pressures, evidenced by reflex activation of the TA muscle (Figure 3).

**Figure 4:**
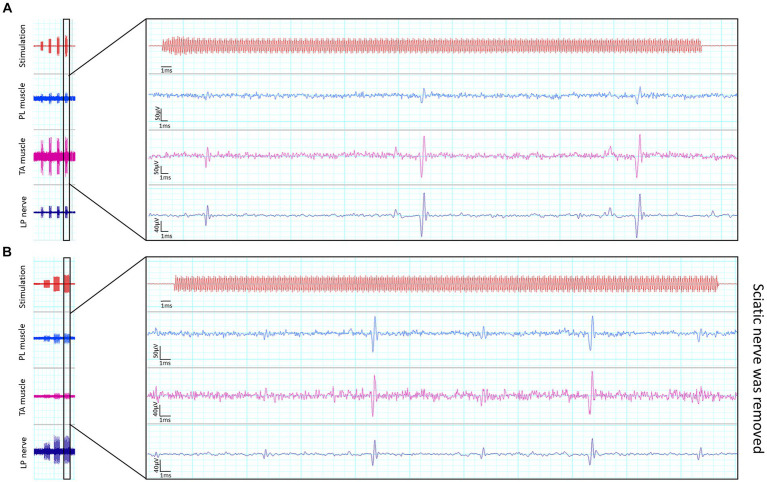
Recordings of artefactual signals. Short duration signals were recorded simultaneously in the three channels during application of FUS **(A)**, even when the sciatic nerve had been removed from the rat **(B)**.

It is worth to note that some artifactual signals were recorded during the FUS application. Some examples are shown in Figure 4. The characteristics of these electrical potentials allow to consider that they are either from non-biological origin, because of too short duration, too low amplitude, and elicited even after dissecting and removing the sciatic nerve in one rat (Figure 4B), or they are spontaneous motor unit action potentials, because they are irregularly firing, not time-locked to FUS and not proportional to intensity of stimulation.

We have also tested under a similar study design whether FUS can activate peripheral motor nerve fibers in the mouse, following the report of Downs et al. (12). Four mice were used, under anesthesia with ketamine-xylazine (90/10 mg/kg). Electrically evoked responses were recorded, however there were no recordable evoked responses elicited by FUS in either muscles or nerves as in the rat (Supplementary Figure S2). To note that FUS delivered by transducer H115 caused macroscopically visible damage in the sciatic nerve of the mouse.

### 3.2 FUS stimulation of peripheral nerves *ex vivo* did not produce action potentials

An *ex vivo* recording setup with the sciatic-tibial nerve of the rat was used for assesing FUS stimulation. Electrical stimulation, applied as a positive control, evoked a CNAP of large amplitude. In contrast, no potentials were recorded following FUS. At high intensity, vibratory motion transmitted within the liquid in the chamber was observed (Supplementary Figure S3).

### 3.3 FUS stimulation induced skin lesions and pain responses

When FUS stimulation was applied noninvasively over the skin at 0.25 and 0.5 MHz, the shaved skin appeared irritated, reddish and inflamed immediately after FUS application, suggesting tissue damage. In contrast, redness was almost imperceptible at 1.63 MHz and absent at 3.58 MHz, indicating that lower US frequencies are more prompt to induce tissue lesions (Supplementary Figure S4). The temperature was measured with a surface thermistor, and found to increase from 25°C to 33–37.5°C near the stimulation spot, more with the 0.25 MHz US than with the higher frequencies.

Withdrawal reflex responses, such as abdominal contraction or paw withdrawal, were observed in some cases when applying US on the skin of the paw. Lower US frequencies induced such pain-induced reactions with less intensity and shorter stimulus duration than higher frequencies. For example, when stimulating at 0.25 MHz at the paw surface, withdrawal reactions could be observed at 1.7 MPa, while higher intensities (3.5 to 5 MPa) were needed to observe them when using 0.5 or 1.63 MHz FUS. The highest frequency tested (3.58 MHz) did not induce any perceptible response in the rats.

### 3.4 Nerve function was affected after exposure to FUS stimulation

**Figure 5:**
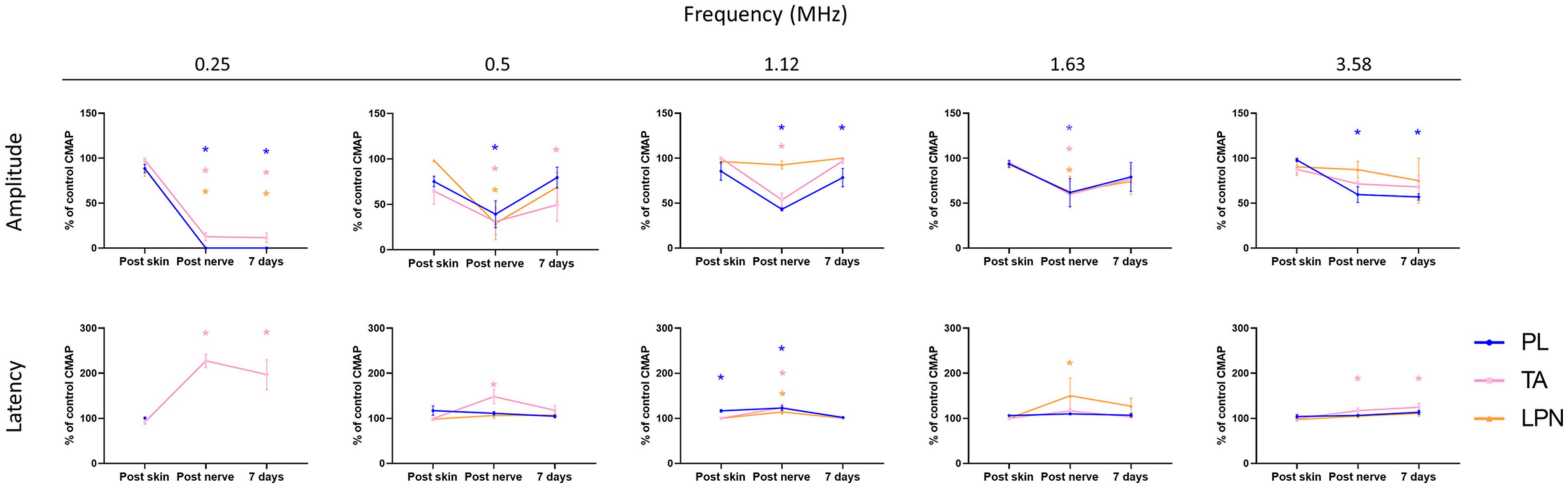
Plots of the PL and TA CMAP and the LPN CNAP amplitude and latency, evoked by electrical stimulation of the proximal sciatic nerve, immediately after the FUS stimulation on the skin, on the exposed sciatic nerve, and one week after the FUS stimulation protocol performed with each one of the transducer frequencies. Values are normalized as percentage of the control baseline values obtained at the beginning of the session. * *p* < 0.05 vs. the control value.

Electrically evoked responses were used to assess nerve functional loss induced by FUS stimulation. After non-invasive FUS stimulation through the skin, the amplitude and latency of the CMAPs and CNAP were similar to control values obtained before stimulation for all the US frequencies applied (Figure 5). However, after stimulating the exposed sciatic nerve, the amplitude of the electrophysiological responses decreased significantly with the transducers of lower frequencies (0.25, 0.5 and 1.12 MHz), and slightly with the high frequencies (1.63 and 3.58 MHz). The latency of the potentials slightly increased after direct nerve stimulation, also indicating involvement of the conduction velocity of impulses. It is worth to note that for the 0.25 MHz stimulation the PL CMAP and the LPN CNAP were abolished in all the rats after stimulation. Nerve conduction properties were still abolished in rats of the 0.25 MHz stimulation after one week, indicating permanent damage to the myelinated fibers in the nerve. On the other hand, the amplitude of the CMAPs and CNAP tended to return toward normal values in rats subjected to FUS stimulation at 0.5, 1.12 and 1.63 MHz. Locomotor function was evaluated with the Walking Track test one week after FUS stimulation. The SFI values indicated that the rats subjected to 0.25 MHz FUS had severe impairment of the motor function (SFI score of −63 ± 3), in line with the affectation of nerve conduction tests, whereas for the other groups the SFI was near normal.

### 3.5 Morphological analysis of the FUS exposed nerves

**Figure 6:**
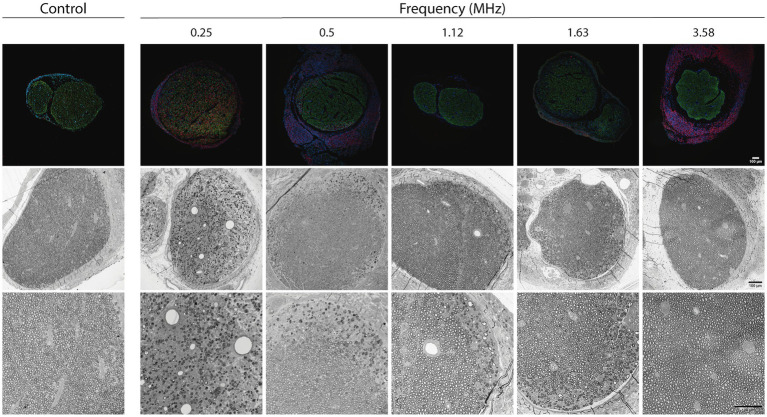
Top images: immunohistochemical images of the sciatic nerve one week after the FUS stimulation protocol performed with each one of the transducer frequencies. Samples were labeled for Neurofilament 200 (RT97, in green), macrophages (Iba1, in red) and nuclei (DAPI, in blue). Mid and bottom images: Representative micrographs of transverse sections of the sciatic nerve one week after the FUS stimulation protocol performed with each one of the FUS frequencies. Note the presence of areas of Wallerian degeneration particularly extense in nerves subjected to FUS at 0.25 and 0.5 MHz. Images of control nerves not subjected to FUS are shown at the left column for comparisons. Bars correspond to 100 μm.

Histomorphological analysis of sciatic nerves was done to control for tissue alterations due to FUS exposure. Samples were harvested 7–9 days after the stimulation to allow sufficient time for damaged nerve fibers to undergo morphological evidence of degeneration. The immunolabeled sciatic nerve cross sections showed maintained architecture of the nerve, with tibial and peroneal fascicles (Figure 6, top panels). Axons, labeled against neurofilaments, were homogeneously distributed. Macrophages, labeled against Iba1, that are scarcely present in control nerves, were largely present in the nerves that had been subjected to FUS stimulation at low frequencies, particularly 0.25 MHz and less 0.5 MHz. This infiltration of inflammatory cells is compatible with the response to nerve fibers injury. Nerves stimulated with 0.25 MHz FUS showed enlarged transverse area due to edema.

Light microscopy of semithin sections of the nerve distal to the FUS application site showed that the architecture of the nerve was preserved in all the rats. The different fascicles were well defined, surrounded by perineurium. For the FUS at frequencies from 0.5 to 3.58 Hz, a normal density of myelinated fibers was observed, with myelin thickness and axon diameter similar to those observed in control nerves (Figure 6, bottom panels). However, in some of the animals (about 50% from each group), some degenerating fibers were observed, characteristically localized at the periphery of the nerve, being the rest of the nerve normal. This axonal degeneration was variable, depending on the nerve and the transducer used, with a tendency to be larger the amount of degeneration with the lower US frequency. In contrast, the transducer with the lowest frequency (0.25 Hz US) produced a severe negative impact on nerve fibers. A high number of degenerative axons and myelin debris were observed, being some nerves almost completely degenerated, whereas other still had some intact myelinated fibers. In this group, at the application site, an important disorganization of the connective tissue could be also observed. The perineurium was disrupted and the different fascicles, with clear signs of degeneration in the endoneurium, were surrounded by a fibrotic reactive tissue.

## 4 Discussion

Medical and research interest on the use of ultrasound neuromodulation is growing in the last years, with potential applications for the non-invasive treatment of neurodegenerative diseases and also for functional tissue imaging (36). Peripheral effects of FUS have been shown to modulate metabolic and immunological responses when applied to the target organs (37). However, direct US activation of axons in peripheral nerves has been investigated in less studies, which reported contradictory results, from enhanced nerve activity, inhibitory effects, and mixed effects. The present study had two main objectives. First, to test whether FUS stimulation is able to activate peripheral nerve axons under different stimulation parameters and conditions, thus being useful as a nerve interface system. Second, to evaluate whether FUS stimulation may inflict damage to the healthy nerve and tissues. The results indicate that FUS stimulation at frequencies between 0.25 and 3.58 MHz cannot directly activate the peripheral nerve fibers in mammals, and that FUS of 0.25–0.5 MHz with pressures below 5 MPa can induce nerve damage after a total accumulated stimulation time of 25,270 ms of FUS stimulation.

We used three setups to assess the effects of FUS stimulation. First, FUS stimulation was applied transcutaneously through the shaved skin of the rat focusing over the sciatic nerve. This setup enables to stimulate the nerve noninvasively, but situates the target a few millimeters below the transducer cone. In a second setup the nerve was surgically exposed, and isolated from surrounding tissues, so that the focus of the transducer was easily placed over the nerve by using a laser pointer, and the vertical focusing distance controlled using a micromanipulator. In previous experimental studies in mammals US stimulation was either applied to the nerves transcutaneously (12, 38, 39) or to exposed nerves but not properly isolated from surrounding tissues (16, 40), which may have confounding effects of stimulation of muscle and skin (14). On the third setup, FUS stimulation was applied on the plantar skin of the paw, over the tibial/sural nerve territory, with the aim of stimulating the nerve fibers either directly or through mechanoreceptors, as reported by Hoffman et al. (15) using an *ex vivo* preparation. A modification of this setup in which the nerve was carefully frayed to increase the number of fibers in contact with the electrodes was also tested. We delivered pulsed FUS stimulation at 0.25, 0.5, 1.12, 1.63 and 3.58 MHz, using parameters ranging from 1 to 10 pulses of 1 to 200 ms and pressure of 0.1 to 5 MPa, and no consistent electrophysiological responses were recorded in any studied setup. Indeed, we applied the largest set of parameters so far examined for US stimulation of peripheral nerves, including pressures below and above levels recommended for safety reasons in diagnostic ultrasound (41).

There are controversial results in the literature about the capability of FUS to directly elicit action potentials in peripheral nerve axons (see reviews by Gavrilov (31, 42)). Previous reports using *in vivo* noninvasive preparations in mice showed recorded muscle action potentials in only a low proportion of cases, and of comparatively very low amplitude compared with electrically evoked potentials (12, 24). Guo et al. (14) demonstrated that US stimulation either transcutaneous or exposing the guinea pig sciatic nerve, with the nerve contacting surrounding tissues, could elicit evoked potentials in the somatosensory cortex, although with very small amplitude. However, when the nerve was fully isolated, such evoked responses disappeared. Of interest, when directly recording from the nerve no CNAPs were noticeable with US stimulation either on the nerve in contact with tissue or isolated. The cortical responses could be, thus, attributable to excitacion of skin or muscle receptors conveying impulses along other nerves in the hindlimb. Our results are in agreement with that study and indicate that US is not able to directly excite the mammalian nerve fibers.

On the other hand, FUS may affect the electrical conductive properties of the peripheral nerves, mainly by the induced changes in focal temperature. It has been found that temperature elevation up to 11.5°C facilitated, but increases above 15°C suppressed the electrically evoked action potentials of motor and sensory nerves (14, 24). When FUS was applied directly to the peripheral nerve *ex vivo*, no action potentials were elicited at any intensity; however, the application of FUS induced a reduction in the latency of electrically produced action potential of A and C fibers, suggesting a neuromodulation effect (28, 40, 43, 44). At relatively high intensity, a partial or complete nerve conduction block was even produced, with recovery taking hours to days (44, 45), an effect that was related to the temperature elevation of the US in some studies but not in others (46). Higher suppression of evoked muscle potentials were found using lower US frequency compared to high frequencies (46). Thus, FUS can safely modulate neural activity in the central and peripheral nervous system and offer new options for noninvasively suppress neural excitation in situations such as pain or spasticity.

In recent reports of *in vivo* US induced peripheral neuromodulation (47, 48), stimulation targets were located directly in end-organs (i.e., neurons, axons, and end-axon terminals within organs). However, in reports that failed to achieve direct FUS-mediated nerve activation, the stimulus target was myelinated and unmyelinated nerve fibers (14, 17–19, 44). In the PNS, cellular components that have been targeted by FUS in the brain (such as cell soma and synaptic connections) are not present in the axonal bundles of the nerve, but in end-organ sites or neural ganglia (37).

In summary, our results show that in our *in vivo* model FUS did not elicit reliable action potentials in either the nerve or the muscle under a wide range of US conditions. Even considering the controversial results published, it does not seem a reliable and safe method for constituting a useful nerve interface for use in humans. We conclude that beyond the stimulation parameters, other conditions appear to affect the peripheral nerve physiological state, determining the possibility of FUS to evoke activity in the healthy peripheral axons. In addition, FUS may cause functional impairment and structural damage to the peripheral nerve, particularly when the nerve is directly exposed to FUS with low frequency, i.e., 0.25 to 0.5 MHz, applied during a few thousand pulses. This deleterious effect should be taken into consideration for potential biomedical applications of FUS.

## Data availability statement

The raw data supporting the conclusions of this article will be made available by the authors, without undue reservation.

## Ethics statement

The animal study was approved by Ethical Committee of the Universitat Autònoma de Barcelona. The study was conducted in accordance with the local legislation and institutional requirements.

## Author contributions

BR-M: Data curation, Formal analysis, Investigation, Validation, Writing – review & editing. ES-N: Investigation, Visualization, Writing – original draft, Methodology. ST-V: Investigation, Visualization, Writing – review & editing, Methodology. AJ: Investigation, Writing – review & editing, Resources, Software, Supervision. EU: Writing – review & editing, Investigation, Data curation, Formal analysis. MF: Conceptualization, Methodology, Resources, Software, Writing – review & editing. XN: Conceptualization, Funding acquisition, Investigation, Methodology, Project administration, Resources, Supervision, Visualization, Writing – original draft, Writing – review & editing.

## Supplementary material

### Supplementary FIGURE 1

**(A)** Experimental setup for investigation of peripheral nerve stimulation on small animals. **(B)** Example of stimulation sequence applied with the 500 kHz transducers consisting of 5 consecutive 20-cycle bursts with increasing amplitude and 100 µs pause between bursts. **(C)** Experimental characterization of the H107 probe driven at 500 kHz. Pressure distribution field measured in a water tank for assessment of the focus position and dimensions. **(D)** Assessment of the focus size in lateral and axial dimension for the case of the H107 transducer driven at 500 kHz. **(E)** Experimental assessment of the pressure versus driving voltage curve for the H107 transducer when driven at different frequencies. The measurement was performed using a calibrated hydrophone positioned in the focal maximum.

### Supplementary FIGURE 2

Representative electrophysiological recordings of CMAPs recorded on plantar interosseus (PL) and tibialis anterior (TA) muscles and of CNAP recorded near the lateral plantar nerve (LPN), evoked by electrical stimulation (ES) of the mouse sciatic nerve, and in response to FUS stimulation (0.25 MHz).

### Supplementary FIGURE 3

Ex vivo assays of FUS stimulation at 0.5 MHz. Recordings obtained evoked by electrical stimulation **(A)** and by FUS stimulation **(B)**. Images of the sciatic nerve placed in a multielectrode chamber **(C)**.

### Supplementary FIGURE 4

Photographs of the skin damage induced by FUS stimulation, when applied through the skin and the paw.

## References

[1] Del Valle J Navarro X. Interfaces with the peripheral nerve for the control of neuroprostheses. Int Rev Neurobiol. (2013) 109:63–83. doi: 10.1016/B978-0-12-420045-6.00002-X, PMID: 24093606

[2] Navarro X Krueger TB Lago N Micera S Stieglitz T Dario P. A critical review of interfaces with the peripheral nervous system for the control of neuroprostheses and hybrid bionic systems. J Peripher Nerv Syst. (2005) 10:229–58. doi: 10.1111/j.1085-9489.2005.10303.x, PMID: 16221284

[3] Schultz AE Kuiken TA. Neural interfaces for control of upper limb prostheses: the state of the art and future possibilities. PM R. (2011) 3:55–67. doi: 10.1016/j.pmrj.2010.06.016, PMID: 21257135

[4] Badia J Boretius T Andreu D Azevedo-Coste C Stieglitz T Navarro X. Comparative analysis of transverse intrafascicular multichannel, longitudinal intrafascicular and multipolar cuff electrodes for the selective stimulation of nerve fascicles. J Neural Eng. (2011) 8:036023. doi: 10.1088/1741-2560/8/3/036023, PMID: 21558601

[5] Badia J Raspopovic S Carpaneto J Micera S Navarro X. Spatial and functional selectivity of peripheral nerve signal recording with the transversal intrafascicular multichannel electrode (TIME). IEEE Trans Neural Syst Rehabil Eng. (2016) 24:20–7. doi: 10.1109/TNSRE.2015.2440768, PMID: 26087496

[6] Anderson JM Rodriguez A Chang DT. Foreign body reaction to biomaterials. Semin Immunol. (2008) 20:86–100. doi: 10.1016/j.smim.2007.11.004, PMID: 18162407 PMC2327202

[7] Branner A Stein RB Fernandez E Aoyagi Y Normann RA. Long-term stimulation and recording with a penetrating microelectrode array in cat sciatic nerve. IEEE Trans Biomed Eng. (2004) 51:146–57. doi: 10.1109/TBME.2003.820321, PMID: 14723504

[8] Christensen MB Pearce SM Ledbetter NM Warren DJ Clark GA Tresco PA. The foreign body response to the Utah slant electrode Array in the cat sciatic nerve. Acta Biomater. (2014) 10:4650–60. doi: 10.1016/j.actbio.2014.07.010, PMID: 25042798

[9] De la Oliva N Del Valle J Delgado-Martinez I Mueller M Stieglitz T Navarro X. Long-term functionality of transversal intraneural electrodes is improved by dexamethasone treatment. IEEE Trans Neural Syst Rehabil Eng. (2019) 27:457–64. doi: 10.1109/TNSRE.2019.2897256, PMID: 30716042

[10] Raspopovic S Capogrosso M Petrini FM Bonizzato M Rigosa J Di Pino G. Restoring natural sensory feedback in real-time bidirectional hand prostheses. Sci Transl Med. (2014) 6:222ra19. doi: 10.1126/scitranslmed.3006820, PMID: 24500407

[11] Shahriari D Rosenfeld D Anikeeva P. Emerging frontier of peripheral nerve and organ interfaces. Neuron. (2020) 108:270–85. doi: 10.1016/j.neuron.2020.09.025 33120023

[12] Downs ME Lee SA Yang G Kim S Wang Q Konofagou EE. Non-invasive peripheral nerve stimulation via focused ultrasound *in vivo*. Phys Med Biol. (2018) 63:035011. doi: 10.1088/1361-6560/aa9fc2, PMID: 29214985

[13] Gavrilov LR Tsirulnikov EM Davies IA. Application of focused ultrasound for the stimulation of neural structures. Ultrasound Med Biol. (1996) 22:179–92. doi: 10.1016/0301-5629(96)83782-3, PMID: 8735528

[14] Guo H Offutt SJ Hamilton Ii M Kim Y Gloeckner CD Zachs DP. Ultrasound does not activate but can inhibit *in vivo* mammalian nerves across a wide range of parameters. Sci Rep. (2022) 12:2182. doi: 10.1038/s41598-022-05226-7, PMID: 35140238 PMC8828880

[15] Hoffman BU Baba Y Lee SA Tong CK Konofagou EE Lumpkin EA. Focused ultrasound excites action potentials in mammalian peripheral neurons in part through the mechanically gated ion channel PIEZO2. Proc Natl Acad Sci USA. (2022) 119:e2115821119. doi: 10.1073/pnas.2115821119, PMID: 35580186 PMC9173751

[16] Juan EJ González R Albors G Ward MP Irazoqui P. Vagus nerve modulation using focused pulsed ultrasound: potential applications and preliminary observations in a rat. Int J Imaging Syst Technol. (2014) 24:67. doi: 10.1002/ima.22080, PMID: 25165410 PMC4142523

[17] Wright CJ Rothwell J Saffari N. Ultrasonic stimulation of peripheral nervous tissue: an investigation into mechanisms. J Phys Conf Ser. (2015) 581:12003. doi: 10.1088/1742-6596/581/1/012003

[18] Tsui P-H Wang S-H Huang C-C. In vitro effects of ultrasound with different energies on the conduction properties of neural tissue. Ultrasonics. (2005) 43:560–565. doi: 10.1016/j.ultras.2004.12.003 15950031

[19] Wright CJ Haqshenas SR Rothwell J Saffari N. Unmyelinated peripheral nerves can be stimulated *in vitro* using pulsed ultrasound. Ultrasound Med Biol. (2017) 43:2269–83. doi: 10.1016/j.ultrasmedbio.2017.05.008, PMID: 28716433

[20] Krasovitski B Frenkel V Shoham S Kimmel E. Intramembrane cavitation as a unifying mechanism for ultrasound-induced bioeffects. Proc Natl Acad Sci USA. (2011) 108:3258–63. doi: 10.1073/pnas.1015771108, PMID: 21300891 PMC3044354

[21] Menz MD Oralkan Ö Khuri-Yakub PT Baccus SA. Precise neural stimulation in the retina using focused ultrasound. J Neurosci. (2013) 33:4550–4560. doi: 10.1523/JNEUROSCI.3521-12.2013 23467371 PMC6704938

[22] Kubanek J Shi J Marsh J Chen D Deng C Cui J. Ultrasound modulates ion channel currents. Sci Reports. (2016) 6:1–14. doi: 10.1038/srep24170 PMC4845013 27112990

[23] Alcaino C Knutson KR Treichel AJ Yildiz G Strege PR Linden DR. A population of gut epithelial enterochromaffin cells is mechanosensitive and requires Piezo2 to convert force into serotonin release. Proc Natl Acad Sci U. S. A. (2018) 115:E7632–E7641. doi: 10.1073/PNAS.1804938115 30037999 PMC6094143

[24] Kim MG Kamimura HAS Lee SA Aurup C Kwon N Konofagou EE. Image-guided focused ultrasound modulates electrically evoked motor neuronal activity in the mouse peripheral nervous system *in vivo*. J Neural Eng. (2020) 17:026026. doi: 10.1088/1741-2552/ab6be6, PMID: 31940596 PMC7297566

[25] Lee SA Kamimura HAS Burgess MT Konofagou EE. Displacement imaging for focused ultrasound peripheral nerve neuromodulation. IEEE Trans Med Imaging. (2020) 39:3391–402. doi: 10.1109/TMI.2020.2992498, PMID: 32406828 PMC7717066

[26] Pasquinelli C Hanson LG Siebner HR Lee HJ Thielscher A. Safety of transcranial focused ultrasound stimulation: a systematic review of the state of knowledge from both human and animal studies. Brain Stimul. (2019) 12:1367–80. doi: 10.1016/j.brs.2019.07.024, PMID: 31401074

[27] Mihran RT Barnes FS Wachtel H. Temporally-specific modification of myelinated axon excitability in vitro following a single ultrasound pulse. Ultrasound Med Biol. (1990) 16:297–309. doi: 10.1016/0301-5629(90)90008-Z 2363236

[28] Ilham SJ Chen L Guo T Emadi S Hoshino K Feng B. *In vitro* single-unit recordings reveal increased peripheral nerve conduction velocity by focused pulsed ultrasound. Biomed Phys Eng Express. (2018) 4:045004. doi: 10.1088/2057-1976/aabef1, PMID: 30410792 PMC6217852

[29] Raspopovic S Carpaneto J Udina E Navarro X Micera S. On the identification of sensory information from mixed nerves by using single channel cuff electrodes. J Neuroeng Rehabil. (2010) 7:17. doi: 10.1186/1743-0003-7-17, PMID: 20423488 PMC2887885

[30] Cutrone A Del Valle J Santos D Badia J Filippeschi C Micera S. A three-dimensional self-opening intraneural peripheral interface (SELINE). J Neural Eng. (2015) 12:16016. doi: 10.1088/1741-2560/12/1/016016 25605565

[31] Feng B Chen L Ilham SJ. A review on ultrasonic neuromodulation of the peripheral nervous system: enhanced or suppressed activities? Appl Sci (Basel). (2019) 9:1637. doi: 10.3390/app9081637, PMID: 34113463 PMC8188893

[32] Naor O Krupa S Shoham S. Ultrasonic neuromodulation. J Neural Eng. (2016) 13:031003. doi: 10.1088/1741-2560/13/3/031003 27153566

[33] Valero-Cabré A Navarro X. H reflex restitution and facilitation after different types of peripheral nerve injury and repair. Brain Res. (2001) 919:302–12. doi: 10.1016/s0006-8993(01)03052-9, PMID: 11701142

[34] Rodríguez FJ Ceballos D Schüttler M Valderrama E Stieglitz T Navarro X. Polyimide cuff electrodes for peripheral nerve stimulation. J Neurosci Methods. (2000) 98:105–18. doi: 10.1016/S0165-0270(00)00192-8, PMID: 10880824

[35] Valero-Cabré A Navarro X. Changes in crossed spinal reflexes after peripheral nerve injury and repair. J Neurophysiol. (2002) 87:1763–71. doi: 10.1152/jn.00305.2001, PMID: 11929897

[36] Rabut C Yoo S Hurt RC Jin Z Li H Guo H. Ultrasound technologies for imaging and modulating neural activity. Neuron. (2020) 108:93–110. doi: 10.1016/j.neuron.2020.09.003, PMID: 33058769 PMC7577369

[37] Cotero V Miwa H Graf J Ashe J Loghin E Di Carlo D. Peripheral focused ultrasound neuromodulation (pFUS). J Neurosci Methods. (2020) 341:108721. doi: 10.1016/j.jneumeth.2020.108721, PMID: 32387189

[38] Casella DP Dudley AG Clayton DB Pope JC 4th Tanaka ST Thomas J. Modulation of the rat micturition reflex with transcutaneous ultrasound. Neurourol Urodyn. (2017) 36:1996–2002. doi: 10.1002/nau.23241, PMID: 28346718

[39] Foley JL Little JW Vaezy S. Image-guided high-intensity focused ultrasound for conduction block of peripheral nerves. Ann Biomed Eng. (2007) 35:109–19. doi: 10.1007/s10439-006-9162-0, PMID: 17072498

[40] Lele PP. Effects of focused ultrasonic radiation on peripheral nerve, with observations on local heating. Exp Neurol. (1963) 8:47–83. doi: 10.1016/0014-4886(63)90008-6

[41] International Electrotechnical Commission; Geneva, Switzerland: 2015. [(accessed on 28 November 2023)]. IEC 60601–2-37:2007+AMD1:2015 CSV Consolidated Version. Medical Electrical Equipment—Part 2–37: Particular Requirements for the Basic Safety and Essential Performance of Ultrasonic Medical Diagnostic and Monitoring Equipment. Available online: https://webstore.iec.ch/publication/22634

[42] Gavrilov LR Tsirulnikov EM. Focused ultrasound as a tool to input sensory information to humans (Review). Acoust Phys (2012) 58:1–21. doi: 10.1134/S1063771012010083/METRICS

[43] Chen L Ilham SJ Guo T Emadi S Feng B. *In vitro* multichannel single-unit recordings of action potentials from mouse sciatic nerve. Biomed Phys Eng Express. (2017) 3:045020. doi: 10.1088/2057-1976/aa7efa, PMID: 29568573 PMC5858727

[44] Colucci V Strichartz G Jolesz F Vykhodtseva N Hynynen K. Focused ultrasound effects on nerve action potential *in vitro*. Ultrasound Med Biol. (2009) 35:1737–47. doi: 10.1016/j.ultrasmedbio.2009.05.002, PMID: 19647923 PMC2752482

[45] Foley JL Little JW Vaezy S. Effects of high-intensity focused ultrasound on nerve conduction. Muscle Nerve. (2008) 37:241–250. doi: 10.1002/MUS.20932 18041054

[46] El Hassan RH Lawand NB Al-Chaer ED Khraiche ML. Frequency dependent, reversible focused ultrasound suppression of evoked potentials in the reflex arc in an anesthetized animal. J Peripher Nerv Syst. (2022) 27:271–82. doi: 10.1111/jns.12512, PMID: 36161403

[47] Cotero V Graf J Miwa H Hirschstein Z Qanud K Huerta TS. Stimulation of the hepatoportal nerve plexus with focused ultrasound restores glucose homoeostasis in diabetic mice, rats and swine. Nat Biomed Eng. (2022) 6:683–705. doi: 10.1038/s41551-022-00870-w, PMID: 35361935 PMC10127248

[48] Zachs DP Offutt SJ Graham RS Kim Y Mueller J Auger JL. Noninvasive ultrasound stimulation of the spleen to treat inflammatory arthritis. Nat Commun. (2019) 10:951. doi: 10.1038/s41467-019-08721-0, PMID: 30862842 PMC6414603

