# Supplementary material for: Assessment of focused ultrasound stimulation to induce peripheral nerve activity and potential damage in vivo
Source: Front Neurol. 2024 Feb 28;15:1346412. doi: 10.3389/fneur.2024.1346412 (PMC10932961; doi:10.3389/fneur.2024.1346412)
Supplement: Supplementary file 1 [file Table_1.docx]

Supplementary Table 1. Protocol for FUS stimulation applied with each transducer at 0.25, 0.5, 1.12, 1.63 and 3.58 MHz.

| Number of sequences | Number of pulses | Pulse duration (ms) | Interstimulus interval (ms) | Intersequence Interval (ms) | Intensities (MPa) |
| --- | --- | --- | --- | --- | --- |
| 5 | 1 | 1 | 100 | 1000 | 0 / 1.25 / 2.5 / 3.75 / 5 |
| 5 | 1 | 10 | 100 | 1000 | 0 / 1.25 / 2.5 / 3.75 / 5 |
| 5 | 1 | 50 | 100 | 1000 | 0 / 1.25 / 2.5 / 3.75 / 5 |
| 5 | 1 | 100 | 100 | 1000 | 0 / 1.25 / 2.5 / 3.75 / 5 |
| 5 | 1 | 200 | 200 | 1000 | 0 / 1.25 / 2.5 / 3.75 / 5 |
| 5 | 3 | 1 | 100 | 1000 | 0 / 1.25 / 2.5 / 3.75 / 5 |
| 5 | 3 | 10 | 100 | 1000 | 0 / 1.25 / 2.5 / 3.75 / 5 |
| 5 | 3 | 50 | 100 | 1000 | 0 / 1.25 / 2.5 / 3.75 / 5 |
| 5 | 3 | 100 | 100 | 1000 | 0 / 1.25 / 2.5 / 3.75 / 5 |
| 5 | 3 | 200 | 200 | 1000 | 0 / 1.25 / 2.5 / 3.75 / 5 |
| 5 | 10 | 1 | 100 | 1000 | 0 / 1.25 / 2.5 / 3.75 / 5 |
| 5 | 10 | 10 | 100 | 1000 | 0 / 1.25 / 2.5 / 3.75 / 5 |
| 5 | 10 | 50 | 100 | 1000 | 0 / 1.25 / 2.5 / 3.75 / 5 |
| 5 | 10 | 100 | 100 | 1000 | 0 / 1.25 / 2.5 / 3.75 / 5 |
| 5 | 10 | 200 | 200 | 1000 | 0 / 1.25 / 2.5 / 3.75 / 5 |
