# Supplementary figures and images for: Assessment of focused ultrasound stimulation to induce peripheral nerve activity and potential damage in vivo
Source: Front Neurol. 2024 Feb 28;15:1346412. doi: 10.3389/fneur.2024.1346412 (PMC10932961; doi:10.3389/fneur.2024.1346412)

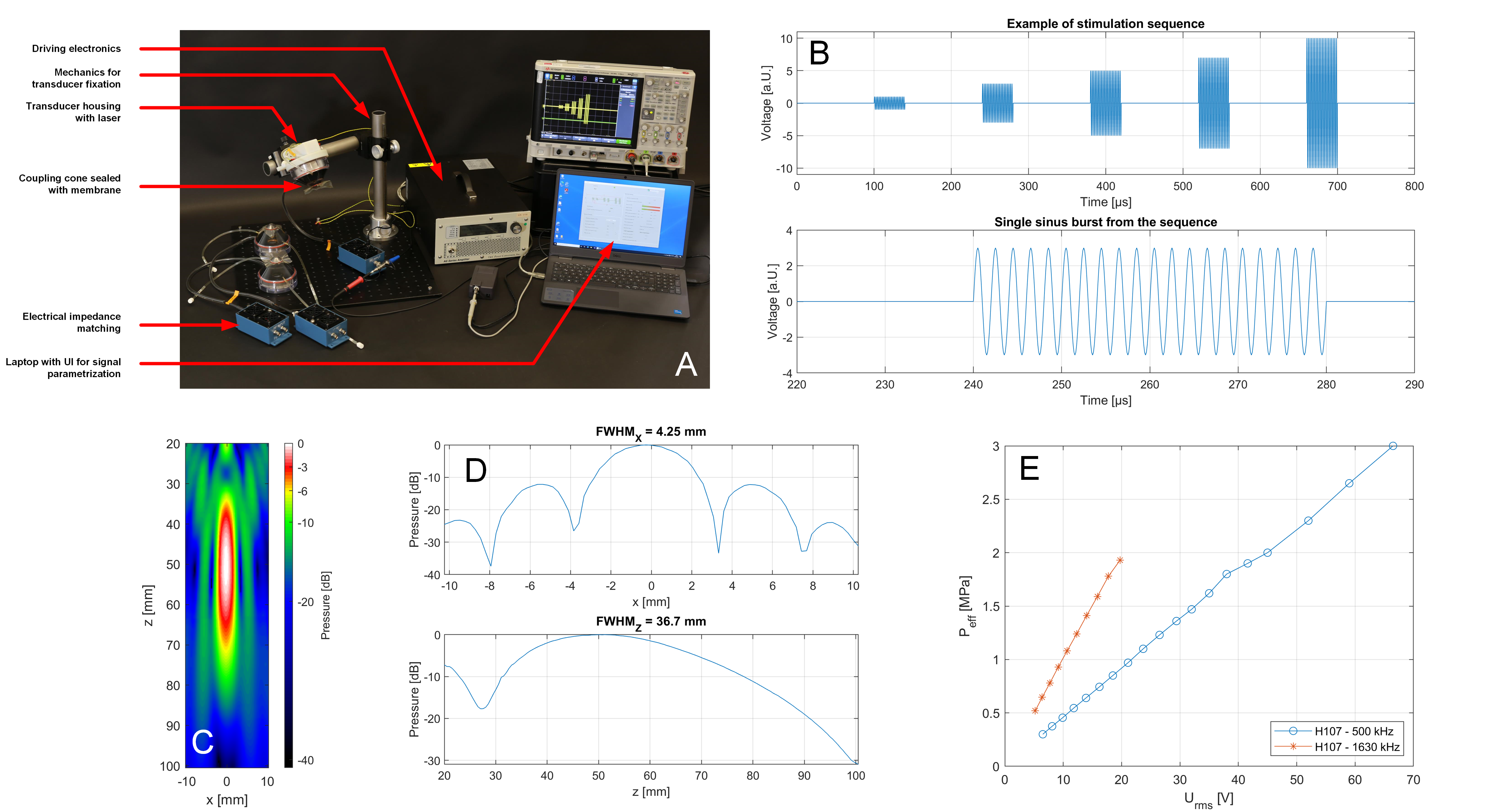

Supplement: Supplementary file 2 [file Image_1.PNG]

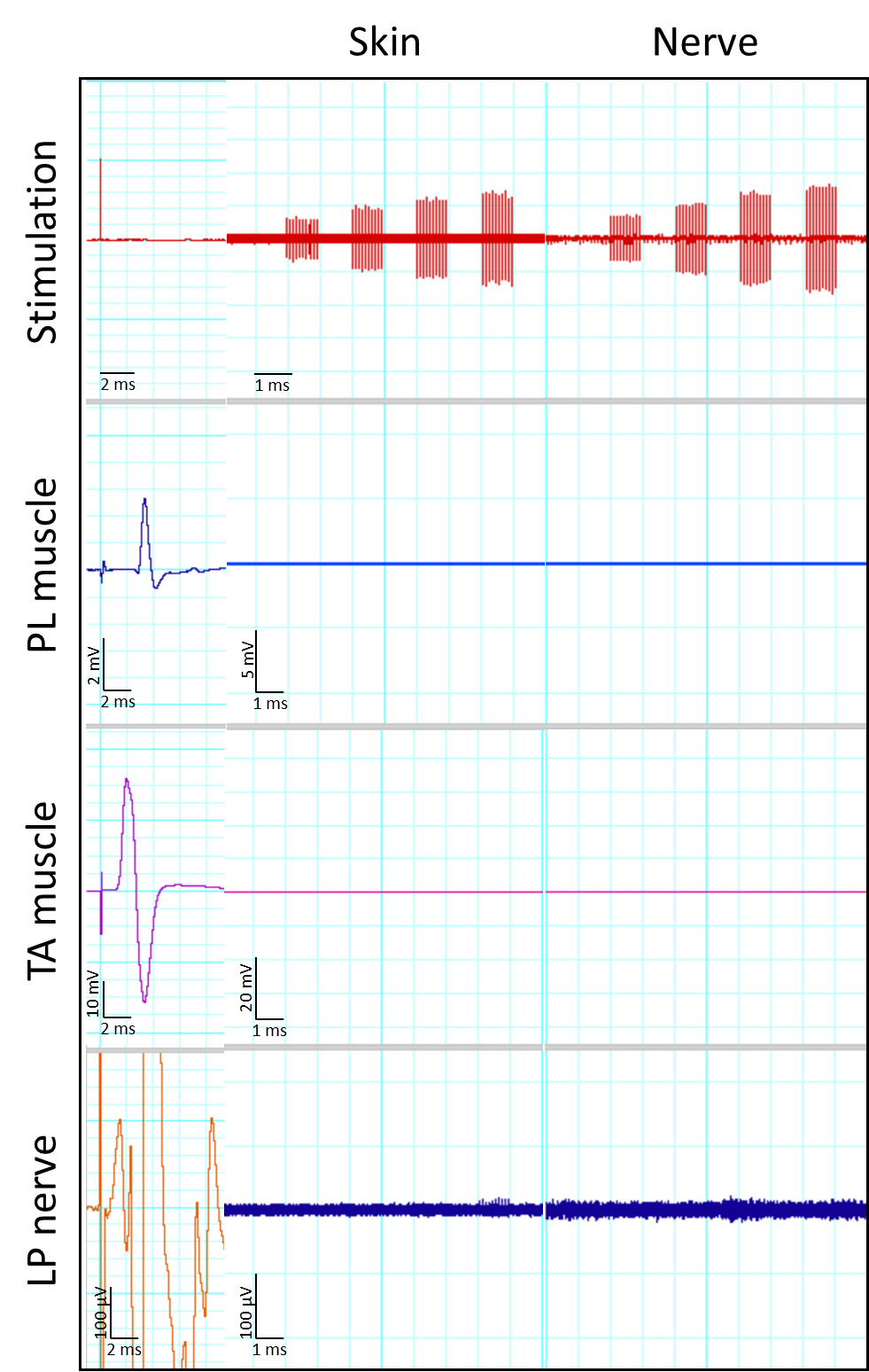

Supplement: Supplementary file 3 [file Image_2.TIF]

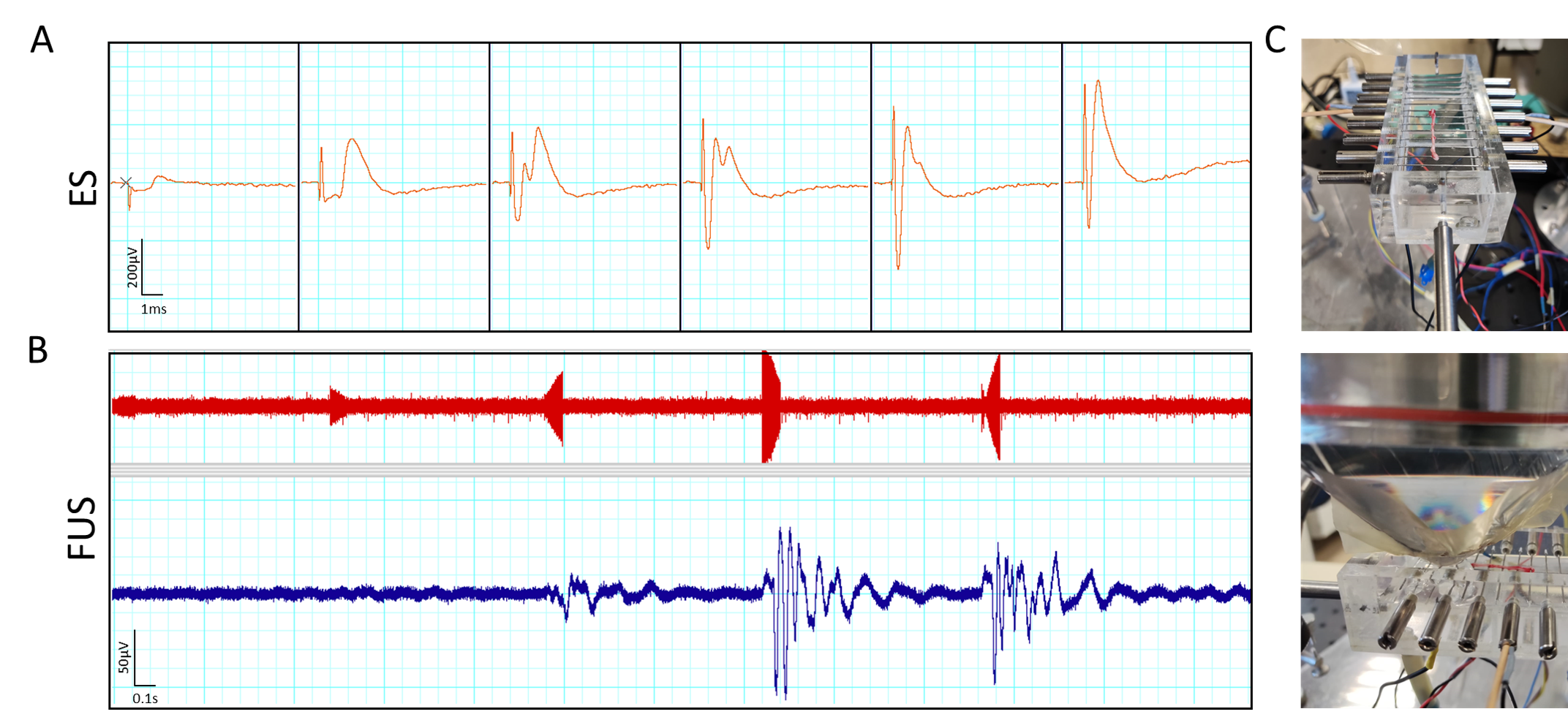

Supplement: Supplementary file 4 [file Image_3.TIF]

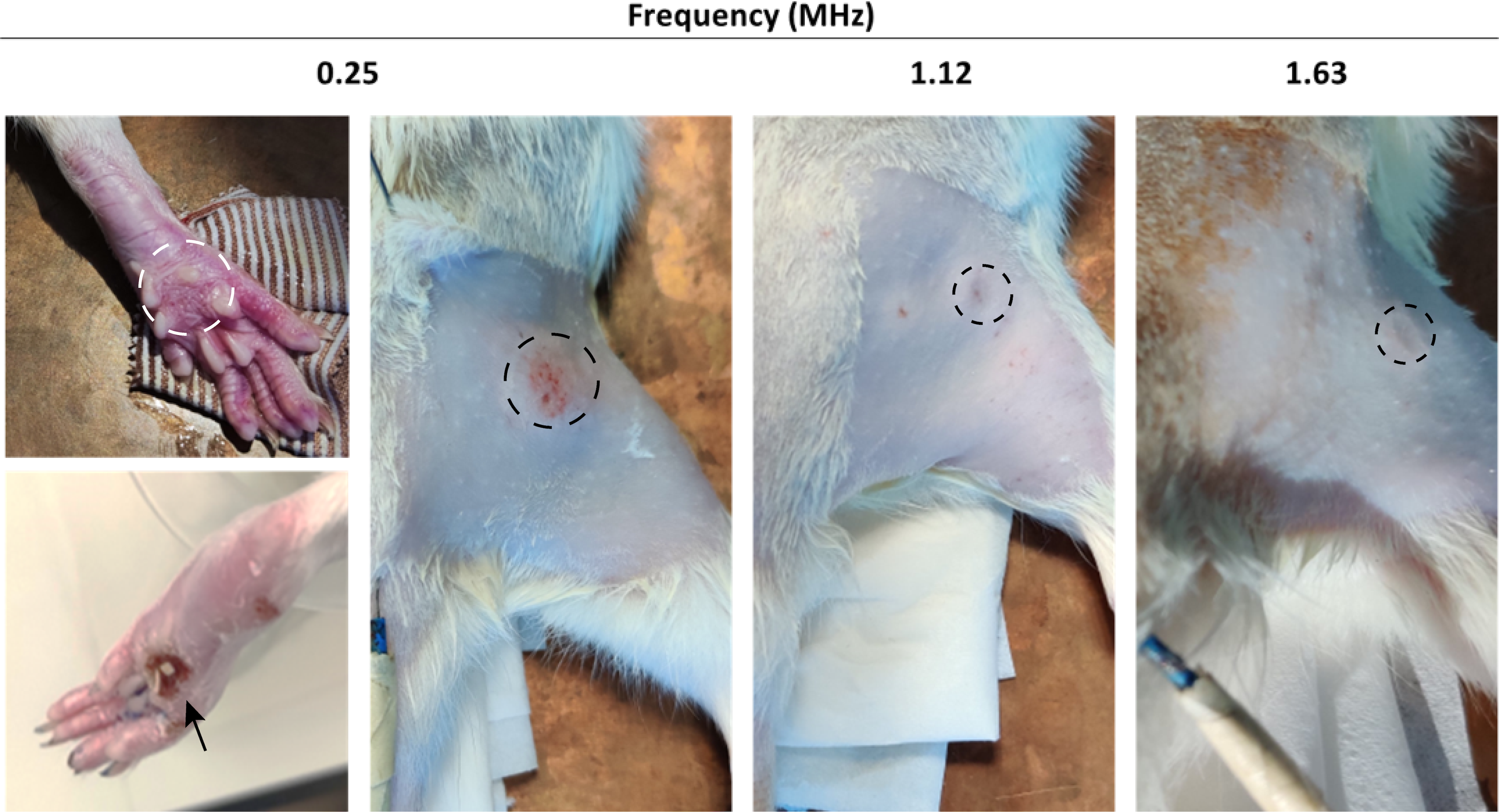

Supplement: Supplementary file 5 [file Image_4.TIF]
